# Bacteria Associated With a Commercial Mycorrhizal Inoculum: Community Composition and Multifunctional Activity as Assessed by Illumina Sequencing and Culture-Dependent Tools

**DOI:** 10.3389/fpls.2018.01956

**Published:** 2019-01-14

**Authors:** Monica Agnolucci, Luciano Avio, Alessandra Pepe, Alessandra Turrini, Caterina Cristani, Paolo Bonini, Veronica Cirino, Fabrizio Colosimo, Maurizio Ruzzi, Manuela Giovannetti

**Affiliations:** ^1^Department of Agriculture, Food and Environment, University of Pisa, Pisa, Italy; ^2^“E. Avanzi” Research Center, University of Pisa, Pisa, Italy; ^3^NGAlab La Riera de Gaia, Tarragona, Spain; ^4^ATENS - Agrotecnologias Naturales SL, La Riera de Gaia, Tarragona, Spain; ^5^Department for Innovation in Biological, Agrofood and Forest Systems, University of Tuscia, Viterbo, Italy

**Keywords:** arbuscular mycorrhizal symbionts, mycorrhizosphere, plant-growth promoting bacteria, siderophores production, indole acetic acid production, metagenomics

## Abstract

The implementation of sustainable agriculture encompasses practices enhancing the activity of beneficial soil microorganisms, able to modulate biogeochemical soil cycles and to affect soil fertility. Among them, arbuscular mycorrhizal fungi (AMF) establish symbioses with the roots of most food crops and play a key role in nutrient uptake and plant protection from biotic and abiotic stresses. Such beneficial services, encompassing improved crop performances, and soil resources availability, are the outcome of the synergistic action of AMF and the vast communities of mycorrhizospheric bacteria living strictly associated with their mycelium and spores, most of which showing plant growth promoting (PGP) activities, such as the ability to solubilize phosphate and produce siderophores and indole acetic acid (IAA). One of the strategies devised to exploit AMF benefits is represented by the inoculation of selected isolates, either as single species or in a mixture. Here, for the first time, the microbiota associated with a commercial AMF inoculum was identified and characterized, using a polyphasic approach, i.e., a combination of culture-dependent analyses and metagenomic sequencing. Overall, 276 bacterial genera were identified by Illumina high-throughput sequencing, belonging to 165 families, 107 orders, and 23 phyla, mostly represented by Proteobacteria and Bacteroidetes. The commercial inoculum harbored a rich culturable heterotrophic bacterial community, whose populations ranged from 2.5 to 6.1 × 10^6^ CFU/mL. The isolation of functional groups allowed the selection of 36 bacterial strains showing PGP activities. Among them, 14 strains showed strong IAA and/or siderophores production and were affiliated with Actinomycetales (*Microbacterium trichotecenolyticum, Streptomyces deccanensis/scabiei*), Bacillales (*Bacillus litoralis, Bacillus megaterium*), Enterobacteriales (*Enterobacter*), Rhizobiales (*Rhizobium radiobacter*). This work demonstrates for the first time that an AMF inoculum, obtained following industrial production processes, is home of a large and diverse community of bacteria with important functional PGP traits, possibly acting in synergy with AMF and providing additional services and benefits. Such bacteria, available in pure culture, could be utilized, individually and/or in multispecies consortia with AMF, as biofertilizers and bioenhancers in sustainable agroecosystems, aimed at minimizing the use of chemical fertilizers and pesticides, promoting primary production, and maintaining soil health and fertility.

## Introduction

Worldwide, a major shift is taking place in agriculture, in order to meet the growing global demand for a safe production of high-quality food, able to maintain or enhance environmental quality and to conserve natural resources for future generations. The implementation of sustainable agriculture encompasses practices enhancing the activity of soil biogeochemical cycles, at the basis of long-term soil productivity and health. The most important players of soil biological fertility are represented by beneficial soil microorganisms, able to modulate biochemical and physiological soil processes, and to affect its biological and nutritional characteristics (Barea et al., 2005). Among them, arbuscular mycorrhizal fungi (AMF, Glomeromycota) are recognized as ecologically and economically important elements of sustainable food production systems, given the key role played in plant nutrition and health, by reducing the input of chemical fertilizers and pesticides (Smith and Read, 2008).

AMF are obligate mutualistic biotrophs, establishing symbioses with the roots of most land plants, including the major food and feed crops, from cereals and legumes to fruits and vegetables, including also important industrial plants, such as sunflower, tobacco, cotton, and medicinal plants (Smith and Read, 2008). AMF symbionts facilitate plant nutrient uptake, mainly phosphorus (P), nitrogen (N), sulfur (S) potassium (K), calcium (Ca), copper (Cu), and zinc (Zn), by means of a large network of extraradical hyphae spreading from colonized roots to the surrounding soil and functioning as a supplementary absorbing system (Giovannetti et al., 2001; Avio et al., 2006). Moreover, they protect plants from biotic and abiotic stresses (Augé, 2001; Evelin et al., 2009; Sikes et al., 2009), provide essential ecosystem services (Gianinazzi et al., 2010), and affect the biosynthesis of beneficial plant secondary metabolites, contributing to the production of safe and high quality food (Sbrana et al., 2014; Avio et al., 2018). However, such beneficial services, encompassing improved crop performances and soil resources availability, are the outcome of the synergistic action of AMF and the vast communities of mycorrhizospheric bacteria living strictly associated with their mycelium and spores (Hildebrandt et al., 2006; Agnolucci et al., 2015). AMF-associated microbiota has been reported to promote mycorrhizal activity (Mayo et al., 1986; Xavier and Germida, 2003; Horii and Ishii, 2006; Giovannetti et al., 2010), to protect plants from soilborne pathogens (Citernesi et al., 1996; Budi et al., 1999; Li et al., 2007; Bharadwaj et al., 2008a,b) and to provide nutrients and growth factors (Barea et al., 2002; Xavier and Germida, 2003), thus being considered as plant growth promoting (PGP) bacteria (PGPB) (Philippot et al., 2013). Molecular investigations allowed the description of the complexity and diversity of bacterial communities associated to AMF spores belonging to different species and isolates, suggesting that their differential occurrence may affect the performance of the relevant taxa in terms of infectivity and efficiency, given their important functional roles as PGPB (Roesti et al., 2005; Long et al., 2008; Agnolucci et al., 2015). Other studies, aimed at isolating and functionally characterizing spore associated bacteria, reported the occurrence of bacteria showing antagonistic activity against plant pathogens (Budi et al., 1999; Bharadwaj et al., 2008a), phosphate-solubilizing and nitrogenase activity (Cruz et al., 2008; Cruz and Ishii, 2011), and indole acetic acid (IAA) production (Bharadwaj et al., 2008a). A recent work, using a culture-dependent approach, showed that bacterial strains isolated in pure culture from *Rhizophagus intraradices* spores were able to solubilize P from phytate and inorganic sources (69.7 and 49.2%, respectively), produce siderophores (65.6%), and IAA (42.6%) (Battini et al., 2016). The last two molecules are very important for plant growth and nutrition. Actually, IAA, a phytohormone of the auxin class, affects the morphology and physiology of roots, enhancing cell division and elongation, and the formation of lateral roots, thus improving water and nutrient uptake and playing a key role in the regulation of plant development (Khalid et al., 2004; Aloni et al., 2006; Duca et al., 2014). Siderophores are low molecular weight, high-affinity iron-chelating compounds able to bind soluble Fe_3_, even at high pH when Fe solubility decreases (Mimmo et al., 2014), thus making it available to bacteria and plants (Colombo et al., 2014). Given the essential role played by iron in plant biochemical processes, such as photosynthesis and respiration (Kobayashi and Nishizawa, 2012), bacterial siderophores, facilitating plant Fe acquisition, represent important factors of plant growth and development (Crowley et al., 1988; Duijff et al., 1994a,b; Walter et al., 1994; Yehuda et al., 1996; Siebner-Freibach et al., 2003; Jin et al., 2006; Vansuyt et al., 2007; Robin et al., 2008). Moreover, siderophores have been reported to possess biocontrol activity against soilborne diseases, by means of iron competition (Thomashow et al., 1990; Glick, 1995; Whipps, 2001), inhibiting the development of deleterious plant pathogens (Davison, 1988; Arora et al., 2001).

Although the individual roles of AMF and their associated bacteria in optimizing plant performance are still to be completely dissected, AMF are progressively more considered among the main factors of sustainable food (primary) production (Philippot et al., 2013; Rouphael et al., 2015). Two main strategies have been devised to exploit the benefits deriving from the mycorrhizal symbionts: the adoption of specific management practices and the use of AMF inoculation. The first one focuses on the improvement of the activity of native AMF, pursued by using crop rotation and mycotrophic cover crops, able to raise soil mycorrhizal potential and to shape native AMF communities (Kabir and Koide, 2002; Karasawa and Takebe, 2012; Lehman et al., 2012; Njeru et al., 2014, 2015; Turrini et al., 2016, 2017), and by reducing tillage intensity or chemical fertilizations, which affect AMF species composition, spore abundance and mycorrhizal colonization (Douds et al., 1995; Jansa et al., 2003; Oehl et al., 2004; Castillo et al., 2006; Brito et al., 2012; Avio et al., 2013). The second strategy focuses on the inoculation of selected AMF, either as single species or in a mixture, reported as efficient root colonizers and plant nutrition enhancers (Jeffries et al., 2003; Gianinazzi and Vosatka, 2004; Lekberg and Koide, 2005; Rouphael et al., 2015).

Many types of commercial AMF inoculum are available on the market, including sterile products obtained *in vitro* using genetically modified Ri T-DNA roots and the species *Rhizoglomus irregulare* (synonym *Rhizophagus irregularis*, basionym *Glomus irregulare*). However, most of the commercial products are obtained from greenhouse multiplication on mycotrophic trap plants and represent a multipartite symbiosis, where a rich community of bacteria may thrive, associated with AMF propagules, and exert important functional activities, as PGPB. Here, for the first time, we explored the bacterial metagenome of a commercially available AMF inoculum by Illumina high-throughput sequencing, a method able to provide information about culturable and unculturable members of the inoculum microbiota. Moreover, we isolated and functionally selected culturable bacteria showing important PGP traits, as the ability to produce IAA and siderophores, to be utilized, individually and/or in multispecies consortia with AMF as beneficial biofertilizers/bioenhancers in sustainable agroecosystems.

## Materials and Methods

### Biological Activity of the Commercial Inoculum

The commercial inoculum utilized consisted of the substrate where trap plants (*Allium ampeloprasum* var. *porrum* L.) were grown and of mycorrhizal root fragments, AMF spores, and extraradical mycelium of *Rhizoglomus irregulare* BEG72 (synonym *Rhizophagus irregularis*, basionym *Glomus irregulare*). The substrate (vermiculite) and the seeds utilized for the inoculum production were sterilized prior to their utilization. The only microbial input in the commercial product arised from the AM fungus and its associated microbiota. The corresponding AMF inoculum is available on the market under the name “AEGIS” (Atens, Agrotecnologias Naturales S.L.). The percentage of mycorrhizal colonization of the roots contained within the inoculum was assessed on three 5 g samples by the gridline intersect method, after clearing with 10% KOH and staining with 0.05% Trypan blue in lactic acid (Giovannetti and Mosse, 1980). The mycorrhizal potential of the commercial inoculum was assessed by using the Mycorrhizal Inoculum Potential (MIP) bioassay, as described in Njeru et al. (2014). Briefly, three replicate inoculum samples were sown with *Cichorium intybus* L. cv. Zuccherina di Trieste, put in sun-transparent bags and maintained in a growth chamber at 27°C and 16/8 h light/dark daily cycle until harvest. Roots were harvested 30 days after sowing, cleaned with tap water and cleared, stained, and examined for AMF colonization assessment, as described above.

### Illumina MySeq Analysis of Bacteria Associated With the Inoculum

#### DNA Extraction

The composition of the bacterial community of three commercial inoculum samples was determined by Next-generation high-throughput DNA sequencing (NGS; Ansorge, 2009). Total community DNA was extracted from each sample using DNeasy PowerSoil Kit (Qiagen, Hilden, Germany). In brief, 50 g of sample and 0.1 mL Tween 20 were suspended in saline phosphate buffer (100 mL) and homogenized in a paddle blender (BagMixer® 400, Interscience, Saint Nom, France) for three min at maximum speed. Substrate soil and root fragments were removed by low speed (1,000 g) centrifugation, then, for DNA extraction, cells were collected after centrifugation and lysed using the DNeasy PowerSoil reagents and Qiagen spin columns on a QIAcube automated station (Qiagen, Hilden, Germany).

#### Library Preparation

Three 16S rRNA gene amplicon libraries were prepared by PCR amplification of an approximate 630 bp region within the hypervariable (V3-V4) region of the 16S rRNA gene according to the Illumina 16S metagenomic sequencing library protocol. PCR amplification was performed with broad spectrum 16S rRNA primers (forward primer: 5′-TCGTCGGCAGCGTCAGATGTGTAT AAGAGACAGCCTACGGGNGGCWGCAG-3′, reverse primer: 5′-GTCTCGTGGGCTCGGAGATGTGTATAAGAGACAGGACTACHVGGGTATCTAATCC-3′) (Klindworth et al., 2013), using Kapa HiFi HotStart 2 × ReadyMix DNA polymerase (Kapa Biosystems Ltd., London, UK). Cycle conditions were: an initial step at 95°C for 3 min; 25 cycles of 95°C (30 s), 55°C (30 s), 72°C (30 s); a final extension of 5 min at 72°C. Libraries were purified using AMPure XP beads (LABPLAN; Naas, Ireland) according to the Illumina 16S metagenomic sequencing library protocol. Dual indices and Illumina sequencing adapters from the Illumina Nextera XT index kits v2 B and C (Illumina, San Diego, USA) were added to the target amplicons in a second Index PCR step using Kapa HotStart HiFi 2 × ReadyMix DNA polymerase (Kapa Biosystems Ltd.). Cycle conditions were: 95°C (3 min); 9 cycles of 95°C (30 s), 55°C (30 s), 72°C (30 s); a final extension of 5 min at 72°C. Libraries were again purified using AMPure XP beads (LABPLAN; Naas, Ireland) according to the Illumina 16S metagenomic sequencing library protocol. Libraries were quantified using a Qubit fluorometer (Life Technologies, Paisley, UK) and pooled in equal concentrations (4 nM) into a single pool, according to their Qubit quantification measurement. The library pool was diluted and denatured according to the Illumina MiSeq library preparation guide. The amplicon library (8 pM) was spiked with 10% denatured and diluted PhiX Illumina control library (12.5 pM). The sequencing run was conducted on the Illumina MiSeq using the 600 cycle MiSeq reagent kit (version 3) with paired 300 bp reads.

#### Sequencing

Illumina sequencing was performed using MiSeq (Illumina, San Diego, CA). Paired-end sequencing used custom primers and a 600-cycle sequencing kit (V3), according to manufacturer instructions. Amplicon sequencing was carried out in the presence of 10% PhiX control (Illumina, San Diego, CA) to allow proper focusing and matrix calculations.

#### Bionformatics

Raw data processing, run de-multiplexing and operational taxonomic unit (OTU) analysis were performed using the CLC Genomics Workbench (Version 11.0.1) with CLC Microbial Genomics Module (Version 3.5) (Qiagen Bioinformatics, Hilden, Germany). Such programme was used also for the estimation of alpha diversity (total richness in OTUs). Taxonomy attribution was performed against SILVA 16S v132 at the identity level of 97%.

### Isolation and Characterization of Beneficial Bacteria Associated With the Inoculum

#### Bacterial Isolation

Three 40 g samples of the commercial inoculum were suspended in 360 mL of sterile physiological solution added with Tween 80 (0.36 μL). The suspension was shaken for 30 min using a multi wrist shaker (Labline Instruments, Illinois, USA). Hundred microliter suspension for each sample were plated in triplicate onto Petri dishes containing different agar media. Culturable heterotrophic bacteria were isolated on Tryptic Soil Agar (TSA, 30 g L^−1^ tryptic soy broth, 20 g L^−1^ bacteriological agar, Oxoid, Milan, Italy), a medium which, given its non-selectivity, allows the recovery of a wide range of aerobic and facultative anaerobic gram-negative and gram-positive bacteria. In order to isolate specific functional bacterial groups, two additional selective media were used. The selective N-free Winogradsky medium (N-free W) (Tchan, 1984) was utilized for the isolation of putative nitrogen-fixing bacteria, able to grow on N-free medium. For the isolation of bacteria able to solubilize inorganic phosphate the National Botanical Research Institute's Phosphate growth (NBRIP) medium was used (Nautiyal, 1999). The three culture media were supplemented with 100 mg L^−1^ of cyclohexymide and 500 UI L^−1^ of nystatin (Sigma–Aldrich, Milan, Italy) to inhibit possible fungal development. The number of colony forming units (CFU) was assessed after 2 and 7 days of incubation at 28°C for TSA and the other two media, respectively. Bacteria grown on N-free W and those showing halo zones formation on NBRIP, were selected and purified by streaking four times onto the same medium used for the isolation. In addition, bacteria grown in TSA medium were randomly selected on the basis of phenotypic colony characteristics, i.e., shape, size, edge morphology, surface and pigment and inoculated onto N-free W and NBRIP media and then purified by streaking four times onto the same medium. The purified strains were maintained at −80°C in cryovials with 20% (v/v) of glycerol in the collection IMA (International Microbial Archives) of the Department of Agriculture, Food and Environment, University of Pisa.

#### Screening of the Selected Bacteria for PGP Traits

**Figure 1:**
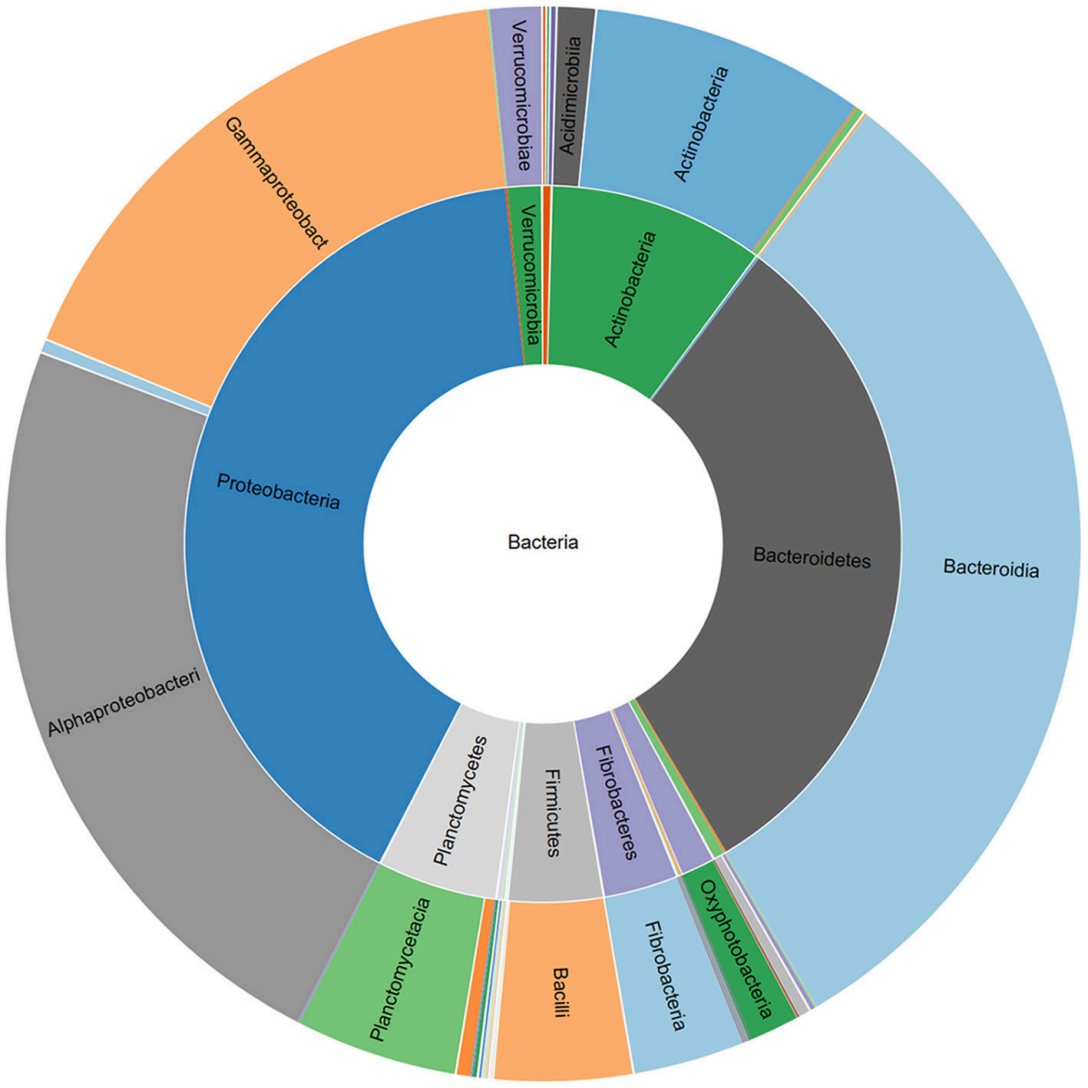
Relative abundance of bacterial phyla and classes associated with a commercial AMF inoculum.

All the bacterial strains isolated and selected as described above, were screened *in vitro* for two functional traits linked to the promotion of plant growth and performance, i. e., the ability to produce IAA and siderophores. The production of IAA was assessed using Luria–Bertani Broth (LBB) (Bharadwaj et al., 2008a) and following the method described by Battini et al. (2016). Briefly, strains were inoculated in 4 mL of LBB amended with 1 mg mL^−1^ of l-tryptophan (Sigma–Aldrich, Milan, Italy), incubated at 20°C until exponential growth phase was reached. They were centrifugated (7500 rpm for 10 min) and 1mL of supernantant was transferred in a 24-well plate, mixed with 2 mL of Salkowski reagent (1.2% FeCl_3_ in 37% sulfuric acid). The non-inoculated medium represented the negative control, and the medium amended with pure IAA the positive one. Development of red–purple color after 3 min incubation in the dark indicated positive strains for IAA production. Strains were classified using a rating scale as follows (Figure 1): –, no production (no color development); +/–, low production (pale pink); +, production (light purple); ++, moderate production (bright purple); +++, high production (dark purple), considering color intensity of the positive controls, IAA (66 μg/mL) representing the maximum value (10+) and IAA 1:2 the half (5+). The test was replicated three times. The ability to produce siderophores was investigated using the overlay Chrome Azurol S assay (CAS) described by Pérez-Miranda et al. (2007). CAS agar was prepared following the procedure described by Louden et al. (2011). Siderophore-producing bacterial strains showed a change in color, from blue to yellow/orange, in the overlaid medium around the colonies. After 7 days the radius of the halo was measured (mm) from the colony edge to the edge of the colored halo. Strains were classified using a rating scale as follows: no production (halo = 0 mm), +/– = low production (halo < 2 mm), + = production (3 mm ≤ halo ≤ 8 mm), ++ = moderate production (9 mm < halo < 14 mm), +++ = high production (halo > 15 mm).

#### Molecular Identification of Cultured PGP Bacteria

The purified bacterial strains showing the best ability to produce IAA and siderophores were identified based on 16S rDNA sequencing. Genomic DNA was extracted from bacterial liquid cultures grown overnight at 28°C using “MasterPure™ Yeast DNA Purification Kit” (Epicenter®), following the manufacturer's protocol. Bacterial 16S rRNA gene was amplified using the primers 27F (5′-GAGAGTTTGATC CTG GCT CAG-3′) e 1495R (5′-CTA CGG CTA CCT TGT TAC GA-3′) (Lane, 1991; Weisburg et al., 1991). The amplification reaction was carried out in a final volume of 25 μL, containing: 5 μL of DyNAzyme buffer 10X (Finnzymes), 0.2 μM of each primer, 0.2 mM of each dNTPs (EuroClone), 0.625 U of Taq DyNAzyme II DNA polymerase (Finnzymes) and 10–20 ng of DNA. The samples were amplified using an iCycleriQ Multicolor Real-Time PCR Detection System (BIORAD), with the following PCR protocol: 95°C 2 min; 94°C 1 min and 20 s, 54°C 1 min, 72°C 1 min, and 30 s for 35 cycles; 72°C 5 min. PCR amplicons were analyzed by 1.5% agarose gel electrophoresis, stained with ethidium bromide, visualized and captured as TIFF format files by the UVITEC UV1-1D program for UVITEC Gel Documentation system Essential V6 (Cambridge, UK). The amplification products were purified by the Clean PCR CleanUp kit” (CABRU), quantified and 5′ sequenced by Eurofins Genomics (Ebersberg, Germany), as reported in Palla et al. (2017). Sequences were analyzed using BLAST on the NCBI web (http://blast.ncbi.nlm.nih.gov/Blast.cgi). The sequences were aligned using MUSCLE, and phylogenetic trees were constructed using the Neighbor-Joining method based on Tamura 3-parameter method in MEGA10 (Kumar et al., 2018) software with 1,000 bootstrap replicates. The sequences were submitted to the European Nucleotide Archive under the accession numbers from LS999506 to LS999519.

## Results

### Biological Activity of the Inoculum

The percentage of colonized length of the root fragments contained within the inoculum was 77±0.7%. The mycorrhizal potential of the inoculum ranged from 20 to 30%.

### Illumina MySeq Analysis of Bacteria Associated With the Inoculum

**Figure 2:**
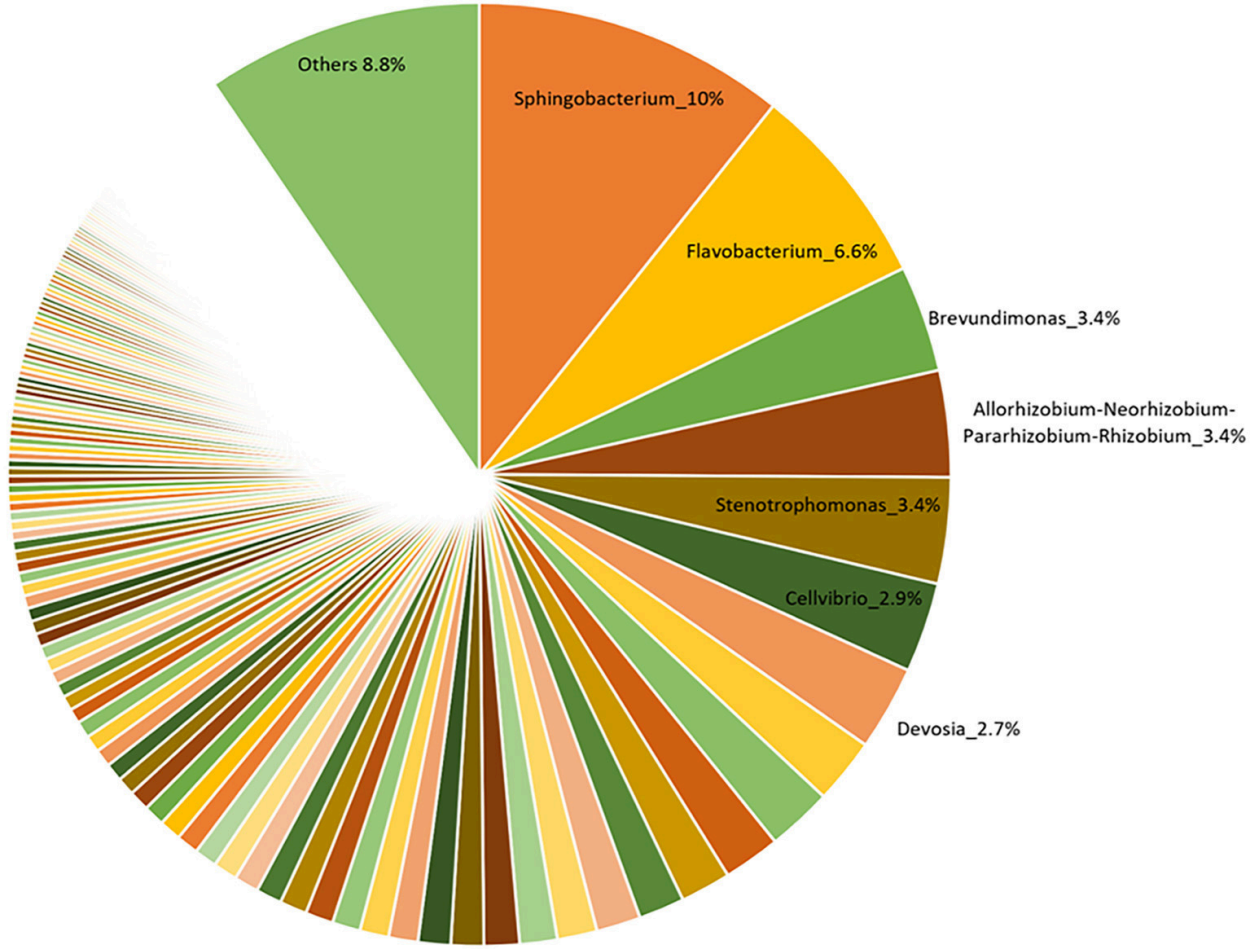
Relative abundance of bacterial genera associated with a commercial AMF inoculum.

The V3-V4 region of 16S rRNA gene was sequenced to analyze the composition of the bacterial microbiota associated with three different lots of AMF inoculum. NGS analysis allowed us to generate a number of reads per sample comprised between 3.1 and 3.9 million (Supplementary Material 1). Approximately 88% of raw reads per sample passed merging, trimming and chimera filtering steps and were analyzed for OTU search. The clustering produced a mean of reads in OTUs of 386,899 ± 25,087 with an average read length after trim of 232 bp. Alpha diversity (OTUs richness) value was 1485 ± 14 (Supplementary Material 1). In total, 23 phyla, 107 orders, 165 families, and 276 bacterial genera were identified in the samples. Nine phyla accounted for 95.8% of the sequence reads across all samples with the majority being Proteobacteria (36.9%) and Bacteroidetes (29.3%; Figure 1). Other phyla that comprised ≥2.5% of the bacterial communities were: Actinobacteria (8.4%), Planctomycetes (6.3%), Verrucomicrobia (3.7%), Firmicutes (3.3%), Patescibacteria (3.1%), Deinococcus-Thermus (2.6%), and Fibrobacteres (2.5%). The predominant orders were: Rhizobiales (23.6%), Caulobacterales (12.9%), Sphingomonadales (12.1%), and Cellvibrionales (9.0%) among Proteobacteria; Sphingobacteriales (45.1%) and Flavobacteriales (34.7%) among Bacteroidetes. A deeper phylogenetic classification of the reads revealed that the most represented genera were *Sphingobacterium* (10% of total bacteria), *Flavobacterium* (6.6%), *Brevundimonas* (3.4%), *Allorhizobium/Neorhizobium/Pararhizobium/Rhizobium* group (3.4%), *Stenotrophomonas* (3.4%), *Cellvibrio* (2.9%), and *Devosia* (2.7%) (Figure 2).

### Isolation and Characterization of Beneficial Bacteria Associated With the Inoculum

Microbiological analyses allowed the determination of the bacterial cells associated with the inoculum. The CFU/ml number of heterotrophic bacteria ranged from 2.5 ± 0.2 to 6.1 ± 1.4 × 10^6^, while putative N-fixers and P-solubilizers ranged from 9.7 ± 0.8 × 10^5^ to 2.2 ± 0.4 × 10^6^ and from 9.2 ± 0.3 × 10^5^ to 2.4 ± 0.9 × 10^6^, respectively (Table 1). A total of 26 putative N-fixers and 9 P-solubilizers were obtained in pure culture. As an additional strain showed both characteristics, the total strains successively tested for their PGP traits were 36.

**Table 1:** Number of culturable bacteria isolated from three 40 g samples (A, B, C) of the AMF commercial inoculum (mean CFU/mL ± SE) isolated from three different microbiological substrates.

**Medium**	**A**	**B**	**C**
TSA	6.1 ±1.4 × 10^6^	3.8 ± 0.6 × 10^6^	2.5 ± 0.2 × 10^6^
N-free W	2.2 ± 0.1 × 10^6^	9.7 ± 0.8 × 10^5^	2.2 ± 0.4 × 10^6^
NBRIP	1.6 ± 0.6 × 10^6^	2.4 ± 0.9 × 10^6^	9.2 ± 0.3 × 10^5^

*Each value represents the mean of three replicates. TSA, Tryptic Soil Agar; N-free W, N-free Winogradsky medium; NBRIP, National Botanical Research Institute's Phosphate growth medium*.

Among the 36 strains analyzed for IAA production, 6 showed the red/orange color similar to the positive controls. Such IAA producers were the isolates N-67 and N-92 within the putative N-fixers, and P-30, P-36, P-42, and P-57 within the P-solubilizers (Supplementary Material 2). The other isolates produced lower levels of IAA, as indicated by the golden yellow color of the substrate (Supplementary Table S1).

As to siderophores production, most strains showed the indicative clarification halo around the colonies, 5 of them producing a halo with a diameter higher than 5 mm, i.e. isolates N-21, N-75, N-78, and N-87 within the putative N-fixers and the isolate P-24 within P-solubilizers (Supplementary Material 2).

**Figure 3:**
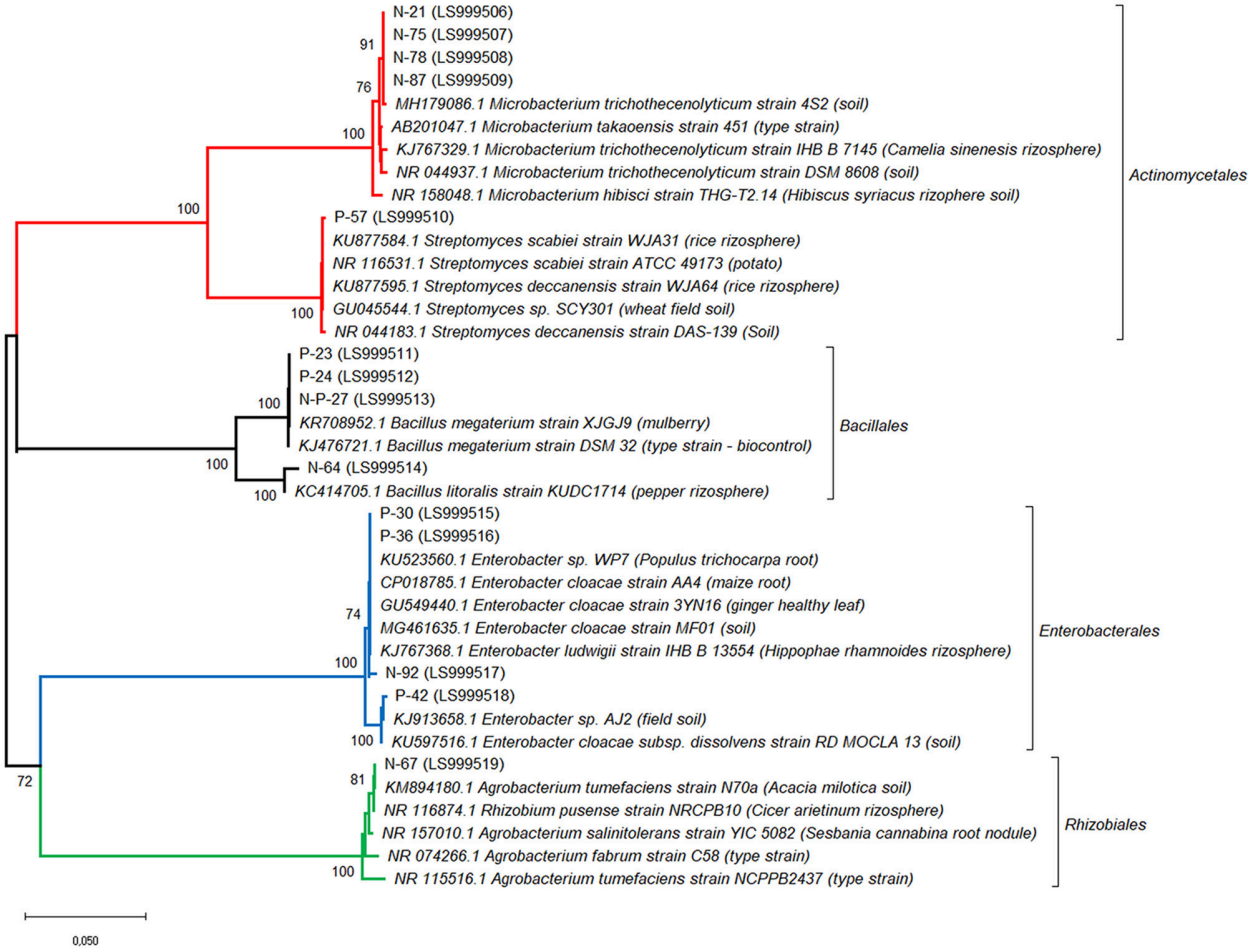
Affiliation of the sequences of the 14 bacterial strains showing the best PGP traits with the existing 16S rRNA gene sequences. Phylogenetic analysis was inferred by using the Neighbor-Joining method. The evolutionary distances were computed using the Tamura 3-parameter method. The rate variation among sites was modeled with a gamma distribution (shape parameter = 1). Bootstrap (1,000 replicates) values below 70 are not shown. Evolutionary analyses were conducted in MEGA10.

Three additional strains, P-23, N-P-27 and N-64, showed a moderate siderophore production, together with IAA production.The 14 bacterial isolates showing the best combination of PGP traits (production of IAA and siderophores) were 16S rDNA sequenced and affiliated to bacterial genera and species. Sequences were affiliated with Actinomycetales (*Microbacterium trichotecenolyticum, Streptomyces deccanensis/scabiei*), Bacillales (*Bacillus litoralis, Bacillus megaterium*), Enterobacteriales (*Enterobacter*), Rhizobiales (*Rhizobium radiobacter*, syn *Agrobacterium radiobacter/tumefaciens*) (Table 2, Figure 3).

**Table 2:** Phylogenetic identification of the 14 best performing plant growth promoting bacteria isolated from the mycorrhizal commercial inoculum (sequence accession numbers from LS999506 to LS999519).

**Isolate**	**Identification**	**Identity (%)**	**Most closely related Genebank sequence**
N-21	*Microbacterium trichotecenolyticum* DSM 8608	99	NR044937
P-23	*Bacillus megaterium* NBRC 15308	99	NR112636
P-24	*Bacillus megaterium* DSM 32	99	KJ476721
N-P-27	*Bacillus megaterium* XJGJ9	99	KR708952
P-30	*Enterobacter* sp. WP7	99	KU523560
P-36	*Enterobacter* sp. WP7	99	KU523560
P-42	*Enterobacter* sp. AJ2	99	KJ913658
P-57	*Streptomyces* sp. SCY301	99	GU045544
N-64	*Bacillus litoralis* KUDC 1714	99	KC414705
N-67	*Rhizobium radiobacter* (syn *Agrobacterium radiobacter*/*tumefaciens*) N70a	99	KM894180
N-75	*Microbacterium trichotecenolyticum* DSM 8608	99	NR044937
N-78	*Microbacterium trichotecenolyticum* DSM 8608	99	NR044937
N-87	*Microbacterium trichotecenolyticum* DSM 8608	99	KJ767329
N-92	*Enterobacter* sp. WP7	99	KU523560

*Strains isolated from N-free Winogradsky and NBRIP, National Botanical Research Institute's Phosphate growth media are preceded by N and P, respectively*.

## Discussion

In this work, for the first time, the microbiota associated with a commercial AMF inoculum was identified and characterized, using a polyphasic approach, i.e., a combination of traditional microbiological culture-dependent analyses and metagenomic sequencing. A complex and highly diverse bacterial community was identified by Illumina high-throughput sequencing and several bacteria showing important PGP traits, as the ability to produce IAA and siderophores, were isolated and identified.

The assessment of mycorrhizal colonization of the roots contained in the inoculum and of the MIP was the necessary prerequisite for the feasibility of our study, given the recent data on the poor colonization of plant roots by a commercial AMF inoculum (Berruti et al., 2013). In our material, both roots contained in commercial inoculum and those of the plants used for the MIP bioassay were well colonized, showing that the commercial inoculum was highly infective and able to rapidly establish the mycorrhizal symbiosis.

The crude inoculum analyzed, consisting of the substrate where trap plants were grown (mycorrhizal root fragments, AMF spores and mycelium) harbored a rich culturable heterotrophic bacterial community, whose populations ranged from 2.5 to 6.1 × 10^6^ CFU/mL. Such values are high, when considering the origin of the sampled material, which did not derive from living roots, but from a dry inoculum, and show that the rich bacterial community thriving in the particular ecological niche, rich in nutrients and exudates, represented by trap plants during AMF inoculum production, is able to maintain its vitality and activity through the different phases leading to the production of the commercial AMF inoculum, from plant harvest to substrate drying. Moreover, present data confirm previous molecular findings which detected large and complex bacterial communities associated with AMF spores (Roesti et al., 2005; Long et al., 2008; Agnolucci et al., 2015).

The culture-independent approach revealed the occurrence of 7 most represented bacterial genera known to include species isolated from a variety of environments that can be subjected to different environmental stresses. For example, bacteria belonging to *Sphingobacterium*, the most represented genus in the commercial inoculum, can survive at temperatures lower than 5°C (Shivaji et al., 1992) and higher than 65°C (Yoo et al., 2007), or can survive in soil contaminated with herbicides (Lü et al., 2006) or solvents (Mohammad et al., 2006). Some species of this genus have been reported to have PGP activities, such as inorganic phosphate solubilization, surfactant and IAA production (Marques et al., 2010; Ahmad et al., 2014; Ali et al., 2017), that can improve the efficacy of AMF inocula. Plant growth-promoting traits were also reported in bacteria belonging to other genera associated with the inoculum, including *Flavobacterium* (phosphate solubilization, production of phytohormones and antimicrobial compounds, Nishioka et al., 2016), *Brevundimonas* (production of IAA and ammonia, Kumar and Gera, 2014), *Stenotrophomonas* (production of antibiotics and plant growth regulators, Messiha et al., 2007) and *Devosia* (development of a nitrogen-fixing root-nodule symbiosis, Rivas et al., 2002). The potential contribution of these bacteria to the efficacy of AMF inocula is supported by recent findings reporting that inoculation with PGPB *Flavobacterium* and *Stenotrophomonas* can be effective in promoting plant growth under draft (Gontia-Mishra et al., 2016) or salinity stress (Singh and Jha, 2017). Interestingly, several sequences (2.9%) were assigned to *Cellvibrio*, a genus known for its cellulose and complex carbohydrate degradation potential, which was previously retrieved from AMF spores, where it was supposed to feed on components of the spore walls, thus facilitating AMF spore germination (Roesti et al., 2005). Many other genera were represented in the bacterial community associated with the commercial inoculum (Supplementary Material 1). Among them, several sequences occurring at low frequencies were ascribed to *Streptomyces* (0.22%), *Enterobacter* (0.24%), *Bacillus* (0.66%), *Microbacterium* (0.83%), genera to which our selected strains belonged.

Here, the inoculation and successive purification on selective media allowed the initial isolation of 36 bacterial strains, and their subsequent screening allowed the selection of the 14 best performing strains showing important PGP traits. Six and five strains were strong producers of IAA and siderophores, respectively, while two of them (N-67 and N-92), displayed at high levels the two PGP traits. The occurrence of such bacterial functional groups in the commercial inoculum further supports our previous evidence that the beneficial microbiota associated with AMF maintains not only its vitality and activity, but also its functional properties during the different phases of the life cycle (Battini et al., 2016). The ability of such strains to produce IAA, a hormone enhancing cell division and boosting the development of plant root systems (Patten and Glick, 2002) and siderophores, able to facilitate plant acquisition of Fe, thus acting as potential biocontrol agents against soilborne plant pathogens (Glick, 1995; Arora et al., 2001; Whipps, 2001; Battini et al., 2016), confirms the need and utility of adopting culture-dependent methods in order to gain knowledge on functional traits of AMF-associated bacteria. The availability of such beneficial bacteria in pure culture allows their use in ecological studies aimed at investigating their mycorrhizospheric competence and role in plant growth promotion.

Fourteen bacterial strains showing the best combination of PGP traits were identified by 16S rDNA sequencing. Interestingly, 5 out of 14 strains (36%) belonged to Actinomycetales: among them, the *Microbacterium trichotecenolyticum* strains N-21, N-75, N-78, N-87, and the *Streptomyces* sp. strain P-57 were strong siderophores and IAA producers, respectively. Actinobacteria are ubiquitous in the soil and able to produce many biologically active secondary metabolites, including antibacterial, antifungal, antiparasitic, anticancer and immunosuppressant drugs (Wolf and Zähner, 1972; Weitnauer et al., 2001; Ritacco and Eveleigh, 2008; Qin et al., 2014) and/or to utilize a wide range of complex compounds (Vandera et al., 2015). They were previously reported to live in strict association with spores and hyphae of different AMF, including *F. coronatum, F. mosseae*, and *R. intraradices* (Walley and Germida, 1996; Andrade et al., 1997; Bharadwaj et al., 2008b; Agnolucci et al., 2015; Battini et al., 2016). Many Actinobacteria showed PGP traits, acting as antagonists against plant pathogens, and mycorrhizal helper traits, enhancing mycorrhizal colonization and AMF functionality (Bharadwaj et al., 2008a; Hamdali et al., 2008; Giovannetti et al., 2010).

Members of the genus *Microbacterium* are ubiquitous in many environments and considered important players of biogeochemical cycles, due to their diazotrophic properties and endophytic behavior (Miliute et al., 2015). Consistent with our findings a *M. trichotecenolyticum* strain isolated from roots of wild *Dodonaea viscosa* L. was reported to possess multiple plant growth promoting activities, such as siderophore and IAA production (Afzal et al., 2017).

The genus *Streptomyces* is one of the main component of soil bacterial communities and is considered within the promising taxa to be investigated for PGP activity, given its ability to solubilize phosphates and produce growth regulators (Mohandas et al., 2013; Hamedi and Mohammadipanah, 2015), two activities shown also by our strain P-57. Actually, two *Streptomyces* strains, W94 and W77, isolated from the spores of the AM fungus *R. irregularis* IMA6, significantly increased the uptake and translocation of ^33^P in maize plants, and hyphal length specific ^33^P uptake, respectively, compared with control plants (Battini et al., 2017). On the other hand, other IAA-producing bacteria isolated from AMF propagules were able to increase AMF development (Bidondo et al., 2011), in agreement with previous data reporting that *Streptomyces* spp. boosted AMF spore germination and hyphal growth (Mugnier and Mosse, 1987; Tylka et al., 1991; Carpenter-Boggs et al., 1995), thus showing mycorrhizal helper traits.

Four out of 14 strains (28%) were affiliated with Bacillales, and belonged to the species *Bacillus megaterium* and *Bacillus litoralis*. All of them produced siderophores, activity previously reported in other members of the order (Battini et al., 2016), known for their ability to control soilborne pathogens (Jeong et al., 2014) and to act as PGP and mycorrhizal helper bacteria, facilitating mycorrhizal establishment and improving plant growth (Budi et al., 2013; Pérez-Montaño et al., 2014; Zhao et al., 2014). The isolation of *Bacillus* species from our commercial inoculum represents a further confirmation of previous data obtained by culture-independent methods (Agnolucci et al., 2015).

One strain, *Rhizobium radiobacter* (syn. *Agrobacterium radiobacter/tumefaciens*) N-67, was affiliated to the Rhizobiales, an order thoroughly investigated for the ability of its members to fix nitrogen. This isolate was one of the two only strains able to produce both IAA and siderophores, confirming previous data on PGP ability of some rhizobia to boost plant nutritional status by producing phytohormones (Zahir et al., 2003; Chandra et al., 2007; Dodd et al., 2010). Its persistence in the AMF inoculum may be ascribed to the formation of biofilms containing exopolysaccharides which allow an efficient colonization of roots and mycorrhizal hyphae (Bianciotto et al., 1996; Toljander et al., 2006).

A very interesting finding is represented by the isolation of 4 strains, P-30, P-36, P-42, N-92, affiliated with Enterobacteriales (*Enterobacter cloacae/ludwigii*), which were strong producers of IAA, confirming previous data on the capacity of a strain of *E. cloacae* to produce as much IAA as a *Pseudomonas* strain (Imen et al., 2013). Recent works reported that a few strains of the genus *Enterobacter*, isolated from legume plants, possessed multiple plant-growth promoting characteristics, such as phosphate solubilisation activity and IAA production, thus affecting plant growth and development (Ghosh et al., 2015; Khalifa et al., 2016). On the other hand, one of our isolates, N-92, produced also siderophores, activity already reported for members of the genus *Enterobacter* (Tian et al., 2009).

In conclusion, this work demonstrates for the first time that an AMF inoculum, produced following industrial production processes, is home of a large and diverse community of bacteria with important functional PGP properties, possibly acting in synergy with AMF and providing new services and benefits. The commercial AMF product could be enriched with the selected beneficial bacterial isolates utilized as an additional inoculum, further boosting plant growth, nutrition and health, in order to optimize plant performance in sustainable food production systems. Indeed, our findings imply a new perspective of AM symbiosis, that of a multipartite association - host plants, AMF and bacteria - where different microbial functional groups are active: for example, specific mycorrhizospheric bacteria, by solubilizing P and fixing N, may improve the availability of key mineral nutrients, then absorbed and translocated to the host plant by AMF extraradical hyphae, while other bacteria, by producing siderophores and IAA, may control plant pathogens and promote plant growth. Notwithstanding, so far only few works have been carried out either on the isolation and functional characterization of mycorrhizospheric microbiota, or on their occurrence and significance in AMF inocula. Yet, these studies are necessary and urgent, in the perspective of developing new strategies for sustainable intensification in agriculture, aimed at minimizing the use of chemical fertilizers and pesticides, promoting primary production and maintaining soil health and fertility. To this aim, the most diverse combinations of AMF and bacteria should be studied, in model experimental systems and in the field, to discover possible synergistic effects on different host plants, in order to select the best performing ones for their targeted use in sustainable food production systems in the years to come.

## Author Contributions

All authors listed have made a substantial, direct and intellectual contribution to the work, and approved it for publication.

### Conflict of Interest Statement

MG received funding from and VC and FC are employees of Atens - Agrotecnologias Naturales SL, La Riera de Gaia, Tarragona, Spain. The remaining authors declare that the research was conducted in the absence of any commercial or financial relationships that could be construed as a potential conflict of interest.

## References

[B1] Afzal I. Iqrar I. Shinwari Z. K. Yasmin A. (2017). Plant growth-promoting potential of endophytic bacteria isolated from roots of wild *Dodonaea viscosa* L. Plant Growth Regul. 81, 399–408. 10.1007/s10725-016-0216-5

[B2] Agnolucci M. Battini F. Cristani C. Giovannetti M. (2015). Diverse bacterial communities are recruited on spores of different arbuscular mycorrhizal fungal isolates. Biol. Fertil. Soils 51, 379–389. 10.1007/s00374-014-0989-5

[B3] Ahmad M. Zahir Z. A. Jamil M. Nazli F. Latif M. Akhtar M. F. (2014). Integrated use of plant growth promoting rhizobacteria, biogas slurry and chemical nitrogen for sustainable production of maize under salt–affected conditions. Pak. J. Bot 46, 375–382.

[B4] Ali L. Khalid M. Asghar H. N. Asgher M. (2017). Scrutinizing of rhizobacterial isolates for improving drought resilience in maize (*Zea mays*). Int. J. Agric. Biol. 19, 1054–1064. 10.17957/IJAB/15.0387

[B5] Aloni R. Aloni E. Langhans M. Ullrich C. I. (2006). Role of cytokinin and auxin in shaping root architecture: regulating vascular differentiation, lateral root initiation, root apical dominance and root gravitropism. Ann. Bot. 97, 883–893. 10.1093/aob/mcl027 16473866 PMC2803412

[B6] Andrade G. Mihara K. L. Linderman R. G. Bethlenfalvay G. J. (1997). Bacteria from rhizosphere and hyphosphere soils of different arbuscular-mycorrhizal fungi. Plant Soil 192, 71–79. 10.1023/A:1004249629643

[B7] Ansorge W. J. (2009). Next-generation DNA sequencing techniques. New Biotechnol. 25, 195–203. 10.1016/j.nbt.2008.12.009 19429539

[B8] Arora N. K. Kang S. C. Maheshwari D. K. (2001). Isolation of siderophore-producing strains of *Rhizobium meliloti* and their biocontrol potential against *Macrophomina phaseolina* that causes charcoal rot of groundnut. Curr. Sci. 81, 673–677.

[B9] Augé R. M. (2001). Water relations, drought and vesicular–arbuscular mycorrhizal symbiosis. Mycorrhiza 11, 3–42. 10.1007/s005720100097

[B10] Avio L. Castaldini M. Fabiani A. Bedini S. Sbrana C. Turrini A. (2013). Impact of nitrogen fertilization and soil tillage on arbuscular mycorrhizal fungal communities in a Mediterranean agroecosystem. Soil Biol. Biochem. 67, 285–294. 10.1016/j.soilbio.2013.09.005

[B11] Avio L. Pellegrino E. Bonari E. Giovannetti M. (2006). Functional diversity of arbuscular mycorrhizal fungal isolates in relation to extraradical mycelial networks. New Phytol. 172, 347–357. 10.1111/j.1469-8137.2006.01839.x 16995921

[B12] Avio L. Turrini A. Giovannetti M. Sbrana C. (2018). Designing the ideotype mycorrhizal symbionts for the production of healthy food. Front. Plant Sci. 9:1089. 10.3389/fpls.2018.01089 30154803 PMC6102486

[B13] Barea J. M. Azcón R. Azcón-Aguilar C. (2002). Mycorrhizosphere interactions to improve plant fitness and soil quality. Anton. Van Leeuw. 81, 343–351. 10.1023/A:1020588701325 12448732

[B14] Barea J. M. Pozo M. J. Azcón R. Aczón-Aguilar C. (2005). Microbial cooperation in the rhizosphere. J. Exp. Bot. 56, 1761–1778. 10.1093/jxb/eri197 15911555

[B15] Battini F. Cristani C. Giovannetti M. Agnolucci M. (2016). Multifunctionality and diversity of culturable bacterial communities strictly associated with spores of the plant beneficial symbiont *Rhizophagus intraradices*. Microb. Res. 183, 68–79. 10.1016/j.micres.2015.11.012 26805620

[B16] Battini F. Grønlund M. Agnolucci M. Giovannetti M. Jakobsen I. (2017). Facilitation of phosphorus uptake in maize plants by mycorrhizosphere bacteria. Sci. Rep. 7:4686. 10.1038/s41598-017-04959-0 28680077 PMC5498536

[B17] Berruti A. Borriello R. Della Beffa M. T. Scariot V. Bianciotto V. (2013). Application of nonspecific commercial AMF inocula results in poor mycorrhization in *Camellia japonica* L. Symbiosis 61, 63–76. 10.1007/s13199-013-0258-7

[B18] Bharadwaj D. P. Lundquist P. O. Alström S. (2008a). Arbuscular mycorrhizal fungal spore-associated bacteria affect mycorrhizal colonization, plant growth and potato pathogens. Soil Biol. Biochem. 40, 2494–2501. 10.1016/j.soilbio.2008.06.012

[B19] Bharadwaj D. P. Lundquist P. O. Persson P. Alström S. (2008b). Evidence for specificity of cultivable bacteria associated with arbuscular mycorrhizal fungal spores. FEMS Microbiol. Ecol. 65, 310–322. 10.1111/j.1574-6941.2008.00515.x 18631178

[B20] Bianciotto V. Bandi C. D. Minerdi M. Sironi H. Tichy V. Bonfante P. (1996). An obligately endosymbiotic mycorrhizal fungus itself harbors obligately intracellular bacteria. Appl. Environ. Microbiol. 62, 3005–3010. 8702293 10.1128/aem.62.8.3005-3010.1996 PMC168087

[B21] Bidondo L. F. Silvani V. Colombo R. Pérgola M. Bompadre J. Godeas A. (2011). Pre-symbiotic and symbiotic interactions between *Glomus intraradices* and two *Paenibacillus* species isolated from AM propagules. *In vitro* and *in vivo* assays with soybean (AG043RG) as plant host. Soil Biol. Biochem. 43, 1866–1872. 10.1016/j.soilbio.2011.05.004

[B22] Brito I. Goss M. J. De Carvalho M. Chatagnier O. van Tuinen D. (2012). Impact of tillage system on arbuscular mycorrhiza fungal communities in the soil under Mediterranean conditions. Soil Tillage Res. 121, 63–67. 10.1016/j.still.2012.01.012

[B23] Budi S. W. Bakhtiar Y. May N. L. (2013). Bacteria associated with arbuscula mycorrhizal spores *Gigaspora margarita* and their potential for stimulating root mycorrhizal colonization and neem (*Melia azedarach* Linn) seedling growth. Microbiol. Indones. 6, 180–188. 10.5454/mi.6.4.6

[B24] Budi S. W. van Tuinen D. Martinotti G. Gianinazzi S. (1999). Isolation from *Sorghum bicolor* mycorrhizosphere of a bacterium compatible with arbuscular mycorrhiza development and antagonistic towardssoil-borne fungal pathogens. Appl. Environ. Microbiol. 65, 5148–5150. 10543835 10.1128/aem.65.11.5148-5150.1999 PMC91693

[B25] Carpenter-Boggs L. Loynachan T. E. Stahl P. D. (1995). Spore germination of *Gigaspora margarita* stimulated by volatiles of soil-isolated actinomycetes. Soil Boil. Biochem. 27, 1445–1451. 10.1016/0038-0717(95)00075-P

[B26] Castillo C. G. Rubio R. Rouanet J. L. Borie F. (2006). Early effects of tillage and crop rotation on arbuscular mycorrhizal fungal propagules in an ultisol. Biol. Fertil. Soils 43, 83–92. 10.1007/s00374-005-0067-0

[B27] Chandra S. Choure K. Dubey R. C. Maheshwari D. K. (2007). Rhizosphere competent *Mesorhizobium loti* MP6 induces root hair curling, inhibits *Sclerotinia sclerotiorum* and enhances growth of Indian mustard (*Brassica campestris*). Braz. J. Microbiol. 38, 124–130. 10.1590/S1517-83822007000100026

[B28] Citernesi A. S. Fortuna P. Filippi C. Bagnoli G. Giovannetti M. (1996). The occurrence of antagonistic bacteria in *Glomus mosseae* pot cultures. Agronomie 16, 671–677.

[B29] Colombo C. Palumbo G. He J. Z. Pinton R. Cesco S. (2014). Review on iron availability in soil: interaction of Fe minerals, plants, and microbes. J. Soil Sed. 14, 538–548. 10.1007/s11368-013-0814-z

[B30] Crowley D. E. Reid C. P. P. Szaniszlo P. J. (1988). Utilization of microbial siderophores in iron acquisition by oat. Plant Physiol. 87, 680–685. 10.1104/pp.87.3.680 16666207 PMC1054820

[B31] Cruz A. F. Horii S. Ochiai S. Yasuda A. Ishii T. (2008). Isolation and analysis of bacteria associated with spores of *Gigaspora margarita*. J. Appl. Microbiol. 104, 1711–1717. 10.1111/j.1365-2672.2007.03695.x 18217929

[B32] Cruz A. F. Ishii T. (2011). Arbuscular mycorrhizal fungal spores host bacteria that affect nutrient biodynamics and biocontrol of soil-borne plant pathogens. Biol. Open 1, 52–57. 10.1242/bio.2011014 23213368 PMC3507164

[B33] Davison J. (1988). Plant beneficial bacteria. Nat. Biotechnol. 6, 282–286. 10.1038/nbt0388-282

[B34] Dodd I. C. Zinovkina N. Y. Safronova V. I. Belimov A. A. (2010). Rhizobacterial mediation of plant hormone status. Ann. Appl. Biol. 157, 361–379. 10.1111/j.1744-7348.2010.00439.x

[B35] Douds D. D. Galvez L. Janke R. R. Wagoner P. (1995). Effect of tillage and farming system upon populations and distribution of vesicular-arbuscular mycorrhizal fungi. Agric. Ecosyst. Environ. 52, 111–118. 10.1016/0167-8809(94)00550-X

[B36] Duca D. Lorv J. Patten C. L. Rose D. Glick B. R. (2014). Indole-3-acetic acid in plant–microbe interactions. Anton. Van Leeuw. 106, 85–125. 10.1007/s10482-013-0095-y 24445491

[B37] Duijff B. Bakker P. A. H. M. Schippers B. (1994a). Ferric pseudobactin 358 as an iron source for carnation. J. Plant Nutr. 17, 2069–2078. 10.1080/01904169409364866

[B38] Duijff B. De Kogel W. J. Bakker P. A. H. M. Schippers B. (1994b). Influence of pseudobactin 358 on the iron nutrition of barley. Soil Biol. Biochem. 26, 1681–1688. 10.1016/0038-0717(94)90321-2

[B39] Evelin H. Kapoor R. Giri B. (2009). Arbuscular mycorrhizal fungi in alleviation of salt stress: a review. Ann. Bot. 104, 1263–1280. 10.1093/aob/mcp251 19815570 PMC2778396

[B40] Ghosh P. K. Sen S. K. Maiti T. K. (2015). Production and metabolism of IAA by *Enterobacter* spp. *(Gammaproteobacteria)* isolated from root nodules of a legume *Abrus precatorius L*. Biocat. Agric. Biotech. 4, 296–303. 10.1016/j.bcab.2015.04.002

[B41] Gianinazzi S. Gollotte A. Binet M. N. van Tuinen D. Redecker D. Wipf D. (2010). Agroecology the key role of arbuscular mycorrhizas in ecosystem services. Mycorrhiza 20, 519–530. 10.1007/s00572-010-0333-3 20697748

[B42] Gianinazzi S. Vosatka M. (2004). Inoculum of arbuscular mycorrhizal fungi for production systems, science meets business. Can. J. Bot. 82, 1264–1271. 10.1139/b04-072

[B43] Giovannetti M. Avio L. Sbrana C. (2010). Fungal spore germination and pre-symbiotic mycelial growth–physiological and genetic aspects, in Arbuscular Mycorrhizas: Physiology and Function, eds Koltai H. Kapulnik Y. (Dordrecht: Springer), 3–32.

[B44] Giovannetti M. Fortuna P. Citernesi A. S. Morini S. Nuti M. P. (2001). The occurrence of anastomosis formation and nuclear exchange in intact arbuscular mycorrhizal networks. New Phytol. 151, 717–724. 10.1046/j.0028-646x.2001.00216.x 33853252

[B45] Giovannetti M. Mosse B. (1980). An evaluation of techniques for measuring vesicular-arbuscular infection in roots. New Phytol. 84, 489–500. 10.1111/j.1469-8137.1980.tb04556.x

[B46] Glick B. R. (1995). The enhancement of plant growth by free-living bacteria. Can. J. Microbiol. 41, 109–117. 10.1139/m95-015

[B47] Gontia-Mishra I. Sapre S. Sharma A. Tiwari S. (2016). Amelioration of drought tolerance in wheat by the interaction of plant growth-promoting rhizobacteria. Plant Biol. 18, 992–1000. 10.1111/plb.12505 27607023

[B48] Hamdali H. Hafidi M. Virolle M. J. Ouhdouch Y. (2008). Growth promotion and protection against damping-off of wheat by two rock phosphate solubilizing actinomycetes in a P-deficient soil under greenhouse conditions. Appl. Soil Ecol. 40, 510–517. 10.1016/j.apsoil.2008.08.001

[B49] Hamedi J. Mohammadipanah F. (2015). Biotechnological application and taxonomical distribution of plant growth promoting actinobacteria. J. Ind. Microbiol. Biotechnol. 42, 157–171. 10.1007/s10295-014-1537-x 25410828

[B50] Hildebrandt U. Ouziad F. Marner F.-J. J. Bothe H. (2006). The bacterium *Paenibacillus validus* stimulates growth of the arbuscular mycorrhizal fungus *Glomus intraradices* up to the formation of fertile spores. FEMS Microbiol. Lett. 254, 258–267. 10.1111/j.1574-6968.2005.00027.x 16445754

[B51] Horii S. Ishii T. (2006). Identification and function of *Gigaspora margarita* growth-promoting microorganisms. Symbiosis 41, 135–141.

[B52] Imen C. F. Manel C. Omar S. Moez J. Salwa H. J. (2013). Phytostabilization of moderate copper contaminated soils using co-inoculation of *Vicia faba* with plant-growth-promoting bacteria. J. Basic Microbiol. 53, 1–9. 10.1002/jobm.201300323 24338717

[B53] Jansa J. Mozafar A. Kuhn G. Anken T. Ruh R. Sanders I. R. (2003). Soil tillage affects the community structure of mycorrhizal fungi in maize roots. Ecol. Appl. 13, 1164–1176. 10.1890/1051-0761(2003)13[1164:STATCS]2.0.CO;2

[B54] Jeffries P. Gianinazzi S. Perotto S. Turnau K. Barea J. M. (2003). The contribution of arbuscular mycorrhizal fungi in sustainable maintenance of plant health and soil fertility. Biol. Fertil. Soils 37, 1–16. 10.1007/s00374-002-0546-5

[B55] Jeong H. Choi S. K. Kloepper J. W. Ryu C. M. (2014). Genome sequence of the plant endophyte *Bacillus pumilus* INR7, triggering induced systemic resistance in field crops. Genome Announc. 2:e01093–1114. 10.1128/genomeA.01093-14 25359912 PMC4214988

[B56] Jin C. W. He Y. F. Tang C. X. Wu P. Zheng S. J. (2006). Mechanisms of microbially enhanced Fe acquisition in red clover (*Trifolium pratense* L.). Plant Cell Environ. 29, 888–897. 10.1111/j.1365-3040.2005.01468.x 17087472

[B57] Kabir Z. Koide R. T. (2002). Effect of autumn and winter mycorrhizal cover crops on soil properties, nutrient uptake and yield of sweet corn in Pennsylvania, USA. Plant Soil 238, 205–215. 10.1023/A:1014408723664

[B58] Karasawa T. Takebe M. (2012). Temporal or spatial arrangements of cover crops to promote arbuscular mycorrhizal colonization and P uptake of upland crops grown after nonmycorrhizal crops. Plant Soil 353, 355–366. 10.1007/s11104-011-1036-z

[B59] Khalid A. Arshad M. Kahir Z. A. (2004). Screening plant growth promoting rhizobacteria for improving growth and yield of wheat. J. Appl. Microbiol. 96, 473–480. 10.1046/j.1365-2672.2003.02161.x 14962127

[B60] Khalifa A. Y. Alsyeeh A. M. Almalki M. A. Saleh F. A. (2016). Characterization of the plant growth promoting bacterium, *Enterobacter cloacae* MSR1, isolated from roots of non-nodulating *Medicago sativa*. Saudi J Biol. Sci. 23, 79–86. 10.1016/j.sjbs.2015.06.008 26858542 PMC4705252

[B61] Klindworth A. Pruesse E. Schweer T. Peplies J. Quast C. Horn M.. (2013). Evaluation of general 16S ribosomal RNA gene PCR primers for classical and next-generation sequencing-based diversity studies. Nucleic Acids Res. 41:e1. 10.1093/nar/gks8 22933715 PMC3592464

[B62] Kobayashi T. Nishizawa N. K. (2012). Iron uptake, translocation, and regulation in higher plants. Ann. Rev. Plant Biol. 63, 131–152. 10.1146/annurev-arplant-042811-105522 22404471

[B63] Kumar S. Stecher G. Li M. Knyaz C. Tamura K. (2018). MEGA X: Molecular evolutionary genetics analysis across computing platforms. Molec. Biol. Evol. 35, 1547–1549. 10.1093/molbev/msy096 29722887 PMC5967553

[B64] Kumar V. Gera R. (2014). Isolation of a multi-trait plant growth promoting *Brevundimonas* sp. and its effect on the growth of Bt-cotton. 3 Biotech 4, 97–101. 10.1007/s13205-013-0126-4 28324462 PMC3909574

[B65] Lane D. J. (1991). 16S/23S rRNA sequencing, in Nucleic Acid Techniques in Bacterial Systematic, eds Stackebrandt E. Goodfellow M. (New York, NY: Wiley*)*, 115–175.

[B66] Lehman R. M. Taheri W. I. Osborne S. L. Buyer J. S. Douds D. D. Jr (2012). Fall cover cropping can increase arbuscular mycorrhizae in soils supporting intensive agricultural production. Appl. Soil Ecol. 61, 300–304. 10.1016/j.apsoil.2011.11.008

[B67] Lekberg Y. Koide R. T. (2005). Is plant performance limited by abundance of arbuscular mycorrhizal fungi? A meta-analysis of studies published between 1988 and 2003. New Phytol. 168, 189–204. 10.1111/j.1469-8137.2005.01490.x 16159333

[B68] Li B. Ravnskov S. Xie G. Larsen J. (2007). Biocontrol of *Pythium* damping-off in cucumber by arbuscular mycorrhiza-associated bacteria from the genus *Paenibacillus*. Biocontrol 52, 863–875. 10.1007/s10526-007-9076-2

[B69] Long L. Zhu H. Yao Q. Ai Y. (2008). Analysis of bacterial communities associated with spores of *Gigaspora margarita* and *Gigaspora rosea*. Plant Soil 310, 1–9. 10.1007/s11104-008-9611-7

[B70] Louden B. C. Haarmann D. Lynne A. M. (2011). Use of blue agar CAS assay for siderophore detection. J. Microbiol. Biol. Ed. 12, 51–53. 10.1128/jmbe.v12i1249 23653742 PMC3577196

[B71] Lü Z. Min H. Li N. Shao T. Ye Y. (2006). Variations of bacterial community structure in flooded paddy soil contaminated with herbicide quinclorac. J. Environ. Sci. Health Part B 41, 821–832. 10.1080/03601230600805873 16893772

[B72] Marques A. P. Pires C. Moreira H. Rangel A. O. Castro P. M. (2010). Assessment of the plant growth promotion abilities of six bacterial isolates using *Zea mays* as indicator plant. Soil Biol. Biochem. 42, 1229–1235. 10.1016/j.soilbio.2010.04.014

[B73] Mayo K. Davis R. E. Motta J. (1986). Stimulation of germination of spores of *Glomus versiforme* by spore-associated bacteria. Mycologia 78, 426–431. 10.2307/3793046

[B74] Messiha N. A. S. Van Diepeningen A. D. Farag N. S. Abdallah S. A. Janse J. D. Van Bruggen A. H. C. (2007). *Stenotrophomonas maltophilia*: a new potential biocontrol agent of *Ralstonia solanacearum*, causal agent of potato brown rot. Eur. J. Plant Phatol. 118, 211–225. 10.1007/s10658-007-9136-6

[B75] Miliute I. Buzaite O. Baniulis D. Stanys V. (2015). Bacterial endophytes in agricultural crops and their role in stress tolerance: a review. Zemdirbyste 102, 465–478. 10.13080/z-a.2015.102.060

[B76] Mimmo T. Del Buono D. Terzano R. Tomasi N. Vigani G. Crecchio C. (2014). Rhizospheric organic compounds in the soil microorganism-plant system: their role in iron availability. Eur. J. Soil Sci. 65, 629–642. 10.1111/ejss.12158

[B77] Mohammad B. T. Wright P. C. Bustard M. T. (2006). Bioconversion of isopropanol by a solvent tolerant *Sphingobacterium mizutae* strain. J. Industr. Microbiol. Biotechnol. 33, 975–983. 10.1007/s10295-006-0143-y 16758171

[B78] Mohandas S. Poovarasan S. Panneerselvam P. Saritha B. Upreti K. K. Kamal R. (2013). Guava (*Psidium guajava* L.) rhizosphere *Glomus mosseae* spores harbor actinomycetes with growth promoting and antifungal attributes. Sci. Hortic. 150, 371–376. 10.1016/j.scienta.2012.11.019

[B79] Mugnier J. Mosse B. (1987). Spore germination and viability of a vesicular arbuscular mycorrhizal fungus, *Glomus mosseae*. Trans. Br. Mycol. Soc. 88, 411–413. 10.1016/S0007-1536(87)80018-9

[B80] Nautiyal C. S. (1999). An efficient microbiological growth medium for screening phosphate solubilizing microorganisms. FEMS Microbiol. Lett. 170, 265–270. 10.1111/j.1574-6968.1999.tb13383.x 9919677

[B81] Nishioka T. Elsharkawy M. M. Suga H. Kageyama K. Hyakumachi M. Shimizu M. (2016). Development of culture medium for the Isolation of *Flavobacterium* and *Chryseobacterium* from rhizosphere soil. Microb. Environ. 31, 104–110. 10.1264/jsme2.ME15144 27098502 PMC4912144

[B82] Njeru E. M. Avio L. Bocci G. Sbrana C. Turrini A. Bàrberi P. (2015). Contrasting effects of cover crops on ‘hot spotș arbuscular mycorrhizal fungal communities in organic tomato. Biol. Fertil. Soils 51, 151–166. 10.1007/s00374-014-0958-z

[B83] Njeru E. M. Avio L. Sbrana C. Turrini A. Bocci G. Bàrberi P. (2014). First evidence for a major cover crop effect on arbuscular mycorrhizal fungi and organic maize growth. Agron. Sustain. Dev. 34, 841–848. 10.1007/s13593-013-0197-y

[B84] Oehl F. Sieverding E. Mäder P. Dubois D. Ineichen K. Boller T.. (2004). Impact of long-term conventional and organic farming on the diversity of arbuscular mycorrhizal fungi. Oecologia 138, 574–583. 10.1007/s00442-003-1458-2 14714172

[B85] Palla M. Cristani C. Giovannetti M. Agnolucci M. (2017). Identification and characterization of lactic acid bacteria and yeasts of PDO Tuscan bread sourdough by culture dependent and independent methods. Int. J. Food Microbiol. 250, 19–26. 10.1016/j.ijfoodmicro.2017.03.015 28364622

[B86] Patten C. L. Glick B. R. (2002). Role of *Pseudomonas putida* indoleacetic acid in development of the host plant root system. Appl. Environ. Microbiol. 68, 3795–3801. 10.1128/AEM.68.8.3795-3801.2002 12147474 PMC124051

[B87] Pérez-Miranda S. Cabirol N. George-Téllez R. Zamudio-Rivera L. S. Fernández F. J. (2007). O-CAS, a fast and universal method for siderophore detection. J. Microbiol. Method 70, 127–131. 10.1016/j.mimet.2007.03.023 17507108

[B88] Pérez-Montaño F. Alías-Villegas C. Bellogín R. A. Del Cerro P. Espuny M. R. Jiménez-Guerrero I.. (2014). Plant growth promotion in cereal and leguminous agricultural important plants: from microorganism capacities to crop production. Microbiol. Res. 169, 325–336. 10.1016/j.micres.2013.09.011 24144612

[B89] Philippot L. Raaijmakers J. M. Lemanceau P. van der Putten W. H. (2013). Going back to the roots: the microbial ecology of the rhizosphere. Nat. Rev. Microbiol. 11, 789–799. 10.1038/nrmicro3109 24056930

[B90] Qin Z. Wang X. Rateb M. E. Ass'ad L. A. Jaspars M. Deng Z.. (2014). Disruption of a methyltransferase gene in actinomycin G gene cluster in *Streptomyces iakyrus* increases the production of phenazinomycin. FEMS Microbiol. Lett. 352, 62–68. 10.1111/1574-6968.12370 24383524

[B91] Ritacco F. V. Eveleigh D. E. (2008). Molecular and phenotypic comparison of phaeochromycin-producing strains of *Streptomyces phaeochromogenes* and *Streptomyces ederensis*. J. Ind. Microbiol. Biotechnol. 35, 931–945. 10.1007/s10295-008-0367-0 18488260

[B92] Rivas R. Velázquez E. Willems A. Vizcaíno N. Subba-Rao N. S. Mateos P. F. (2002). A new species of *Devosia* that forms a unique nitrogen-fixing root-nodule symbiosis with the aquatic legume *Neptunia natans* (Lf) Druce. Appl. Environ. Microbiol. 68, 5217–5222. 10.1128/AEM.68.11.5217-5222.2002 12406707 PMC129907

[B93] Robin A. Vansuyt G. Hinsinger P. Meyer J. M. Briat J. F. Lemanceau P. (2008). Iron dynamics in the rhizosphere: consequences for plant health and nutrition. Adv. Agron. 99, 183–225. 10.1016/S0065-2113(08)00404-5

[B94] Roesti D. Ineichen K. Braissant O. Redecker D. Wiemken A. Aragno M. (2005). Bacteria associated with spores of the arbuscular mycorrhizal fungi *Glomus geosporum* and *Glomus constrictum*. Appl. Environ. Microbiol. 71, 6673–6679. 10.1128/AEM.71.11.6673-6679.2005 16269696 PMC1287740

[B95] Rouphael Y. Franken P. Schneider C. Schwarz D. Giovannetti M. Agnolucci M. (2015). Arbuscular mycorrhizal fungi act as biostimulants in horticultural crops. Sci. Hortic. 196, 91–108. 10.1016/j.scienta.2015.09.002

[B96] Sbrana C. Avio L. Giovannetti M. (2014). Beneficial mycorrhizal symbionts affecting the production of health-promoting phytochemicals. Electrophoresis 35, 1535–1546. 10.1002/elps.201300568 25025092

[B97] Shivaji S. Ray M. K. Rao N. S. Saisree L. Jagannadham M. V. Kumar G. S. (1992). Sphingobacterium antarcticus sp. nov., a psychrotrophic bacterium from the soils of *Schirmacher Oasis*, Antarctica*. Int. J. Sys. Evol.Microbiol* 42, 102–106. 10.1099/00207713-42-1-102

[B98] Siebner-Freibach H. Hadar Y. Chen Y. (2003). Siderophores sorbed on Ca-montmorillonite as an iron source for plants. Plant Soil 251, 115–124. 10.1023/A:1022984431626

[B99] Sikes B. A. Kottenie K. Klironomos J. N. (2009). Plant and fungal identity determines pathogen protection of plant roots by arbuscular mycorrhizas. J. Ecol. 97, 1274–1280. 10.1111/j.1365-2745.2009.01557.x

[B100] Singh R. P. Jha P. N. (2017). The PGPR *Stenotrophomonas maltophilia* SBP-9 augments resistance against biotic and abiotic stress in wheat plants. Front. Microbiol. 8:1945. 10.3389/fmicb.2017.01945 29062306 PMC5640710

[B101] Smith S. E. Read D. J. (2008). Mycorrhizal Symbiosis. London: Academic Press.

[B102] Tchan Y. T. (1984). Azotobacteraceae, in Bergey's Manual of Systematic Bacteriology, vol. 1, eds Krieg N. Holt J. G. (London: Williams and Wikins), 219–225.

[B103] Thomashow L. S. Weller D. M. Bonsall R. F. Pierson L. S. (1990). Production of the antibiotic phenazine-1-carbox-ylic acid by fluorescent *Pseudomonas* species in the rhizosphere of wheat. Appl. Environ. Microbiol. 56, 908–912. 16348176 10.1128/aem.56.4.908-912.1990 PMC184320

[B104] Tian F. Ding Y. Zhu H. Yao L. Du B. (2009). Genetic diversity of siderophore-producing bacteria of tobacco rhizosphere. Braz. J. Microbiol. 40, 276–284. 10.1590/S1517-83822009000200013 24031358 PMC3769708

[B105] Toljander J. F. Artursson V. Paul L. R. Jansson J. K. Finlay R. D. (2006). Attachment of different soil bacteria to arbuscular mycorrhizal fungal extraradical hyphae is determined by hyphal vitality and fungal species. FEMS Microbiol. Lett. 254, 34–40. 10.1111/j.1574-6968.2005.00003.x 16451176

[B106] Turrini A. Caruso G. Avio L. Gennai C. Palla M. Agnolucci M. (2017). Protective green cover enhances soil respiration and native mycorrhizal potential compared with soil tillage in a high-density olive orchard in a long term study. Appl. Soil Ecol. 116, 70–78. 10.1016/j.apsoil.2017.04.001

[B107] Turrini A. Sbrana C. Avio L. Njeru E. M. Bocci G. Bàrberi P. (2016). Changes in the composition of native root arbuscular mycorrhizal fungal communities during a short-term cover crop-maize succession. Biol. Fertil. Soils 52, 643–653. 10.1007/s00374-016-1106-8

[B108] Tylka G. L. Hussey R. S. Roncadori R. W. (1991). Axenic germination of vesicular–arbuscular mycorrhizal fungi: effects of selected *Streptomyces* species. Phytopathology 81, 754–759.

[B109] Vandera E. Samiotaki M. Parapouli M. Panayotou G. Koukkou A. I. (2015). Comparative proteomic analysis of *Arthrobacter phenanthrenivorans* Sphe3 on phenanthrene, phthalate and glucose. J. Proteomics 113, 73–89. 10.1016/j.jprot.2014.08.018 25257624

[B110] Vansuyt G. Robin A. Briat J. Curie C. Lemanceau P. (2007). Iron acquisition from Fe-pyoverdine by *Arabidopsis thaliana*. Mol. Plant-Micr. Interac. 20, 441–447. 10.1094/MPMI-20-4-0441 17427814

[B111] Walley F. L. Germida J. J. (1996). Failure to decontaminate *Glomus clarum* NT4spores is due to spore wall-associated bacteria. Mycorrhiza 6, 43–49. 10.1007/s005720050104

[B112] Walter A. Römheld V. Marschner H. Crowley D. E. (1994). Iron nutrition of cucumber and maize: effect of *Pseudomonas putida* YC3 and its siderophore. Soil Biol. Biochem. 26, 1023–1031. 10.1016/0038-0717(94)90117-1

[B113] Weisburg W. G. Barns S. M. Pelletier D. A. Lane D. J. (1991). 16S ribosomal DNA amplification for phylogenetic study. J. Bacteriol. 173, 697–703. 10.1128/jb.173.2.697-703.1991 1987160 PMC207061

[B114] Weitnauer G. Mühlenweg A. Trefzer A. Hoffmeister D. Süssmuth R. D Jung G.. (2001). Biosynthesis of the orthosomycin antibiotic avilamycin A: deductions from the molecular analysis of the avi biosynthetic gene cluster of *Streptomyces viridochromogenes* Tü57 and production of new antibiotics. Chem. Biol. 8, 569–581. 10.1016/S1074-5521(01)00040-0 11410376

[B115] Whipps J. M. (2001). Microbial interactions and biocontrol in the rhizosphere. J. Exp. Bot. 52, 487–511. 10.1093/jexbot/52.suppl_1.487 11326055

[B116] Wolf H. Zähner H. (1972). Metabolic products of microorganism. Arch. Mikrobiol. 83, 147–154. 4554808

[B117] Xavier L. J. C. Germida J. J. (2003). Bacteria associated with *Glomus clarum* spores influence mycorrhizal activity. Soil Biol. Biochem. 35, 471–478. 10.1016/S0038-0717(03)00003-8

[B118] Yehuda Z. Shenker M. Romheld V. Marschner H. Hadar Y. Chen Y. (1996). The role of ligand exchange in the uptake of iron from microbial siderophores by gramineous plants. Plant Physiol. 112, 1273–1280. 10.1104/pp.112.3.1273 12226445 PMC158055

[B119] Yoo S. H. Weon H. Y. Jang H. B. Kim B. Y. Kwon S. W. Go S. J.. (2007). Sphingobacterium composti sp. nov., isolated from cotton-waste composts. Int. J. Sys. Evol.Microbiol. 57, 1590–1593. 10.1099/ijs.0.64948-0 17625199

[B120] Zahir Z. A. Arshad M. Frankenberger W. T. Jr. (2003). Plant growth promoting rhizobacteria: applications and perspectives in agriculture, in Advances in Agrononomy vol. 81, ed Sparks D. L. (Newark, NY: Academic Press), 97–168.

[B121] Zhao L. Wu X. Q. Ye J. R. Li H. Li G. E. (2014). Isolation and characterization of a mycorrhiza helper bacterium from rhizosphere soils of poplar stands. Biol. Fertil. Soils 50, 593–601. 10.1007/s00374-013-0880-9

