# Supplementary material for: Bacteria Associated With a Commercial Mycorrhizal Inoculum: Community Composition and Multifunctional Activity as Assessed by Illumina Sequencing and Culture-Dependent Tools
Source: Front Plant Sci. 2019 Jan 14;9:1956. doi: 10.3389/fpls.2018.01956 (PMC6339933; doi:10.3389/fpls.2018.01956)
Supplement: Supplementary file 2 [file Table_2.DOCX]

Supplementary Material

Bacteria associated with a commercial mycorrhizal inoculum: community composition and multifunctional activity as assessed by Illumina sequencing and culture-dependent tools

**Monica Agnolucci^1^, Luciano Avio^1^, Alessandra Pepe^1^, Alessandra Turrini^1^, Caterina Cristani^2^, Paolo Bonini^3^, Veronica Cirino^4^, Fabrizio Colosimo^4^, Maurizio Ruzzi^5^, Manuela Giovannetti^1*^**

*** Correspondence:** Manuela Giovannetti: manuela.giovannetti@unipi.it

**Supplementary Table S2.** Functional traits of the 36 PGP Bacteria isolated from the commercial inoculum. Strains isolated from N-free Winogradsky and NBRIP, National Botanical Research Institute’s Phosphate growth media are preceded by N and P, respectively.

| **Isolate** | **IAA production** | | **Siderophore production Activity (after 7 days)** |
| --- | --- | --- | --- |
| N-1 | | ± | **++** |
| N-18 | | **+** | **+** |
| N-21 | | **-** | **+++** |
| P-23 | | **+** | **++** |
| P-24 | | **+** | **+++** |
| N-P-27 | | **+** | **++** |
| P-30 | | **++++** | **-** |
| P-34 | | ± | **-** |
| P-35 | | **-** | **-** |
| P-36 | | **+++** | **-** |
| P-39 | | ± | **-** |
| P-42 | | **++++** | **-** |
| P-57 | | **++++** | **-** |
| N-64 | | **+** | **++** |
| N-65 | | **±** | **++** |
| N-66 | | **-** | **++** |
| N-67 | | **+++** | **++** |
| N-68 | | **±** | **++** |
| N-69 | | **±** | **++** |
| N-70 | | **-** | **++** |
| N-73 | | **±** | **++** |
| N-74 | | **±** | **++** |
| N-75 | | **+** | **+++** |
| N-77 | | **±** | **++** |
| N-78 | | **+** | **+++** |
| N-79 | | **±** | **++** |
| N-80 | | **±** | **++** |
| N-83 | | **+** | **+** |
| N-84 | | **±** | **++** |
| N-85 | | **±** | **++** |
| N-87 | | **±** | **+++** |
| N-88 | | **±** | **++** |
| N-89 | | **±** | **++** |
| N-90 | | **±** | **++** |
| N-91 | | **±** | **++** |
| N-92 | | **++++** | **++** |

Nitrogen fixation: − = absence of fixation, + = N fixation. Phosphate solubilization: − = absence of solubilization, + = solubilization. IAA production: − = no production, +/− = low production, + = production, ++ = moderate production, +++ = high production. Siderophore producton: − = no production (halo=0 cm), **±** = low production (halo ≤ 0.2 cm), + = production (0.3 cm ≤ halo ≤ 0.8 cm), ++ = moderate production (0.9 cm < halo < 1.4 cm), +++ = high production (halo > 1.5 cm).


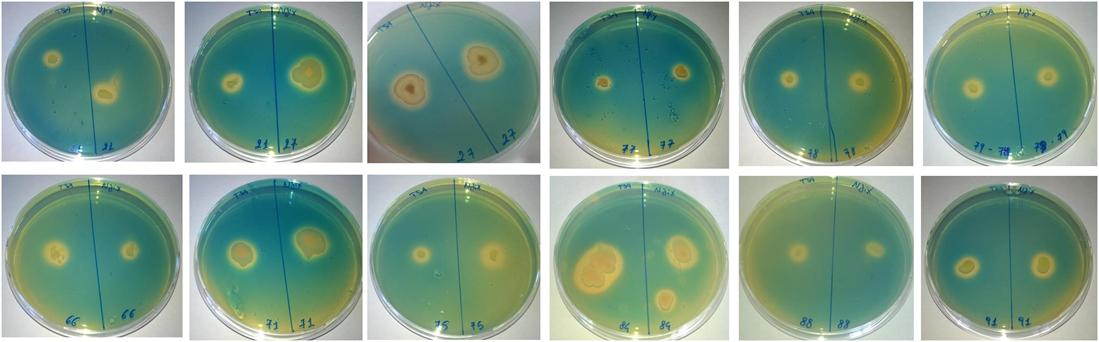


**Supplementary Figure S2.** O-CAS assay for siderophere production showing the halo zone formed around colonies of different bacterial strains isolated from the mycorrhizal commercial inoculum.
